# Snail regulates Nanog status during the epithelial–mesenchymal transition via the Smad1/Akt/GSK3β signaling pathway in non-small-cell lung cancer

**DOI:** 10.18632/oncotarget.2006

**Published:** 2014-05-26

**Authors:** Chen-Wei Liu, Ching-Hao Li, Peng Yi-Jen, Yu-Wen Cheng, Huei-Wen Chen, Po-Lin Liao, Jaw-Jou Kang, Mao-Hsiung Yeng

**Affiliations:** ^1^ Graduate Institute of Medical Science, National Defense Medical Center, Taipei, Taiwan; ^2^ Department of Physiology, School of Medicine, College of Medicine, Taipei Medical University, Taipei, Taiwan; ^3^ Graduate Institute of Medical Sciences, College of Medicine, Taipei Medical University, Taipei, Taiwan; ^4^ Department of Pathology, Tri-Service General Hospital, National Defense Medical Center, Taipei, Taiwan; ^5^ School of Pharmacy, Taipei Medical University, Taipei, Taiwan; ^6^ Institute of Toxicology, College of Medicine, National Taiwan University, Taipei, Taiwan; ^7^ Department of Pharmacology, National Defense Medical Center, Taipei, Taiwan

**Keywords:** Snail, Nanog, epithelial–mesenchymal transition, cancer stem cell, Smad, Akt, Noggin, non-small-cell lung cancer

## Abstract

The epithelial–mesenchymal transition (EMT), a crucial step in cancer metastasis, is important in transformed cancer cells with stem cell-like properties. In this study, we established a Snail-overexpressing cell model for non-small-cell lung cancer (NSCLC) and investigated its underlying mechanism. We also identified the downstream molecular signaling pathway that contributes to the role of Snail in regulating Nanog expression. Our data shows that high levels of Snail expression correlate with metastasis and high levels of Nanog expression in NSCLC. NSCLC cells expressing Snail are characterized by active EMT characteristics and exhibit an increased ability to migrate, chemoresistance, sphere formation, and stem cell-like properties. We also investigated the signals required for Snail-mediated Nanog expression. Our data demonstrate that LY294002, SB431542, LDN193189, and Noggin pretreatment inhibit Snail-induced Nanog expression during EMT. This study shows a significant correlation between Snail expression and phosphorylation of Smad1, Akt, and GSK3β. In addition, pretreatment with SB431542, LDN193189, or Noggin prevented Snail-induced Smad1 and Akt hyperactivation and reactivated GSK3β. Moreover, LY294002 pretreatment prevented Akt hyperactivation and reactivated GSK3β without altering Smad1 activation. These findings provide a novel mechanistic insight into the important role of Snail in NSCLC during EMT and indicate potentially useful therapeutic targets for NSCLC.

## INTRODUCTION

Lung cancer is the most common cause of cancer mortality in the world, with an estimated annual 1.3 million deaths worldwide [1]. It is often diagnosed at an advanced stage and has a poor prognosis. This poor prognosis is due not only to late diagnoses but also to a high rate of recurrence, against which chemotherapy and radiotherapy have limited efficacy. Non-small-cell lung cancer (NSCLC), like nearly 90% of human cancers, is epithelial in origin, and the increased motility and invasiveness of cancer cells are reminiscent of the epithelial–mesenchymal transition (EMT). Accumulating evidence suggests that EMT can occur during tumor progression, constituting an early step in the metastasis of tumors from their primary sites [2]. A recent report also suggests that there may be a direct link between EMT and the acquisition by cells of stem cell-like properties [3]. Such cells have been denoted as cancer-initiating cells or cancer stem cells (CSCs) [4]. The findings from these earlier studies suggest that cells that undergo EMT gain stem cell-like signatures and that CSCs exhibit a mesenchymal-like appearance in several cancer cell types [5].

Recent studies have indicated that the Snail protein is sometimes associated with invasion, metastasis, and/or poor prognosis [6]. Snail can be induced by many tumor-stimulating cytokines such as transforming growth factor beta (TGF-β)/bone morphogenetic protein (BMP) family members, Wnt, and Notch [7-9] and can be negatively regulated by glycogen synthase kinase-3 beta (GSK3β) [10]. Activation of the phosphatidylinositol-3 kinase (PI-3 kinase)/Akt pathway is one of the key signals in EMT and is subject to TGF-β/BMP regulation [11]. Importantly, the PI-3 kinase/Akt pathway can cooperate with TGF-β/BMP signaling to induce EMT. These observations suggest a link between Snail and EMT. Cancer cells with ectopic Snail expression have been shown to induce EMT, although the signaling pathway and downstream effectors responsible for this are unclear. Additionally, associations between EMT and CSCs induced by Snail have not been previously examined. Thus, the detailed molecular signaling pathway underlying the regulatory link between stem-cell related genes and Snail during EMT is still poorly understood.

Here, we investigated the biological significance of increased Snail expression in cultured NSCLC cells and found that cells acquire Nanog expression after EMT through a Snail-dependent mechanism. Snail-overexpressing transfectants exhibit expansion of a CD44^high^/CD24^low^ subpopulation with increased surface CD133 (CSC biomarker) expression, an EMT phenotype, and Smad1/Akt/GSK3β pathway activation. Snail knockdown, Smad1 knockdown, or Smad1/Akt inhibition significantly reduced Nanog expression and rescued GSK3β activity. Taken together, our results indicate that Nanog expression via the activation of the Smad1/Akt/GSK3β pathway is required to sustain the cancer stem cell-like traits generated by Snail-induced EMT.

## RESULTS

### Snail expression is correlated with human NSCLC malignancy

**Figure 1:**
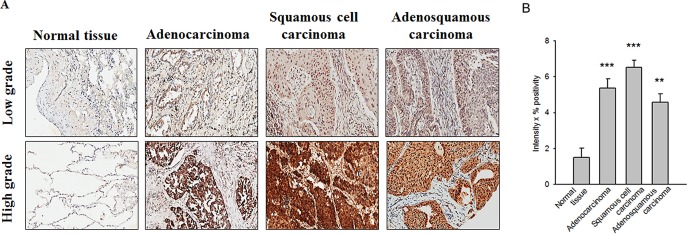
Snail upregulation is correlated with the malignancy of human non-small-cell lung cancer (NSCLC) tissues (A) Representative images of immunohistochemical staining of Snail in specimens from 55 NSCLC patients. In different tumor types (normal tissue, adenocarcinoma, squamous cell carcinoma, and adenosquamous carcinoma), the expression level of Snail was obvious in high-grade but not low-grade NSCLC tumors. (B) The expression level of Snail was analyzed and quantified by an experienced pathologist; **p < 0.01 and ***p < 0.001 indicate statistical significance as compared to the control (normal tissue).

First, we examined Snail reactivity in lung cancer tissues of 40 male and 15 female patients. Tumor clinicopathological features (including cell type, staging, and differentiation) are summarized in Table 1. Snail expression patterns in normal tissue, adenocarcinoma, squamous cell carcinoma, and adenosquamous carcinoma were detected by immunohistochemistry staining. Representative images and quantified data are presented in Figure 1. We found that Snail was mainly expressed in nuclei and that Snail expression showed significant correlation with age (*p* = 0.041), cell type (*p* = 0.039), clinicopathological grade (*p* = 0.012), and tumor status (*p* = 0.0429; Table 1), indicating that Snail has a critical role in directing tumors toward malignancy.

**Table 1:** Correlation between Snail, Nanog expression and clinicopathological characteristics of lung cancer

Snail Expression				
	n	Positive (%)	Negative (%)	P Value
Total	55	34 (62)	21 (38)	
Age				
> 60	22	10 (44)	12 (56)	0.041
<60	33	24 (63)	9 (27)	
Gender				
Male	40	26 (63)	14 (34)	0.4203
Female	15	8 (53)	7 (47)	
Cell type				
AD	14	5 (36)	9 (64)	0.039
SQ	31	24 (77)	7 (23)	
AS	7	3 (43)	4 (57)	
MA	3	2 (67)	1 (33)	
Grade				
1	13	6 (46)	7 (54)	0.012
2	27	14 (52)	13(48)	
3	15	14 (93)	1 (7)	
TMN				
T2	51	31 (61)	20 (39)	0.573
T3	4	3 (75)	1 (25)	
N0	38	22 (58)	16 (42)	0.5660
N1	12	9 (75)	3 (25)	
N2	5	3 (60)	2 (40)	
M0	52	31 (60)	21 (40)	0.04292
M1	3	3 (100)	0 (0)	

### Snail overexpression induces EMT in A549 and CL1-5 NSCLC

**Figure 2:**
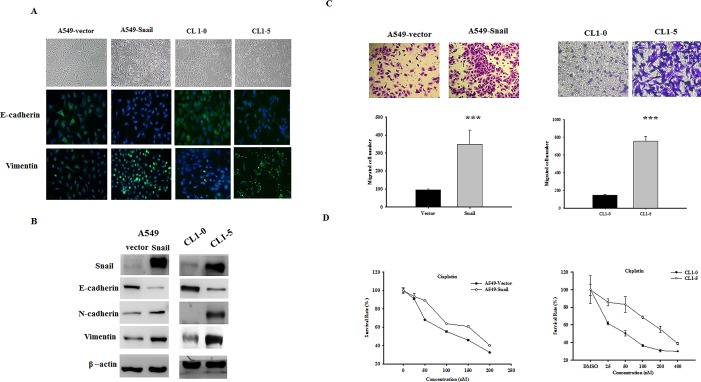
Snail overexpression induces the epithelial–mesenchymal transition (EMT) in A549 and CL1-5 cells (A) Phase-contrast images present the morphology of A549 cells expressing empty vector (A549-vector cells), A549 cells overexpressing Snail (A549-Snail cells), CL1-0 cells, and CL1-5 cells. Immunofluorescent staining of E-cadherin and vimentin (green fluorescence) show the changes in EMT biomarkers. Nuclei were stained with Hoechst 33342 (blue fluorescence). (B) Western blots showing the expression of epithelial markers and mesenchymal markers in A549-vector cells, A549-Snail cells, CL1-0 cells, and CL1-5 cells. In Snail-expressing cells, expression of mesenchymal markers increased, but expression of epithelial markers decreased. (C) The number of migrating cells was significantly increased in Snail-expressing cells; ***p < 0.001 indicates statistical significance as compared to the control. (D) Chemoresistance as evaluated by the MTT assay. The LC_50_ for cisplatin in A549-vector and A549-Snail cells was 134.6 nM and 170.3 nM, respectively. The LC_50_ for cisplatin in CL1-0 and CL1-5 cells was 148.4 nM and 287.6 nM, respectively; CL1-5 is more resistant to cisplatin than CL1-0.

To study the role of Snail in EMT cells with stem cell-like properties, a human Snail cDNA construct was transfected into the A549 NSCLC cell line, which lacks endogenous Snail protein expression. CL1-0 cells (which have a typical epithelial morphology) and CL1-5 cells (which have a mesenchymal, fibroblast-like morphology) are NSCLC cell lines that were generated from the same patient and classified by their metastatic profile [12]. CL1-5 cells have endogenous Snail expression, whereas CL1-0 cells only weakly express Snail. In comparison to the controls (A549 cells transfected with empty vector [A549-vector cells] and CL1-0 cells), Snail-expressing cells (A549-Snail cells) and CL1-5 cells were dispersed, had reduced cell–cell contact, and exhibited an elongated, fibroblast-like morphology (Figure 2A). Immunofluorescent staining and western blotting revealed that the mesenchymal markers vimentin and N-cadherin were upregulated, whereas the epithelial marker E-cadherin was downregulated in Snail-overexpressing cells as compared to control cells (Figure 2A, B). In addition, the number of migrating cells in Snail-expressing cells (A549-Snail cells and CL1-5 cells) was significantly higher than that in the control cells (A549-vector cells and CL1-0 cells), which is indicative of increased invasiveness (Figure 2C). Evaluation of anticancer drug resistance revealed that the LC_50_ of cisplatin for the A549-vector and A549-Snail cells was 134.6 nM and 170.3 nM, respectively. Our data also show that A549-Snail cells are significantly more resistant than the A549-vector cells to SAHA and LBH589 (Supplementary Figure 1). In comparison to CL1-0 cells, CL1-5 cells are significantly more motile and more resistant to cisplatin treatment (Figure 2D).These data show that EMT does occur in Snail-expressing cells.

### Overexpressing Snail promotes in vivo metastatic and tumorigenic abilities in A549 cells

**Figure 3:**
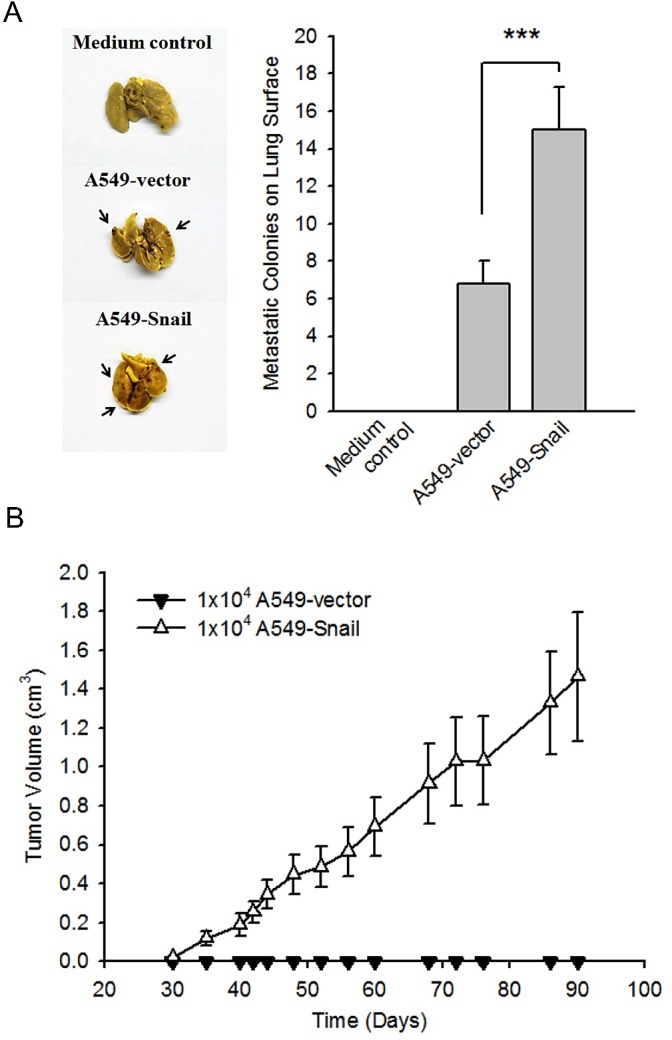
Overexpression of Snail enhances *in vivo* metastatic and tumorigenic abilities in A549 cells (A) The *in vivo* pulmonary metastatic colonies assay was performed as described in the Methods section. Both the images and the analyzed data (N = 5) demonstrate the aggressive metastatic capacity of A549 cells overexpressing Snail (A549-Snail cells) as compared to A549 cells expressing empty vector (A549-vector cells); ***p < 0.001 indicates statistical significance as compared to the A549-vector cells. (B) A549-vector cells or A549-Snail cells (1 × 10^4^ cells) were injected into the subrenal space in NOD/SCID mice. The growth curves of xenograft tumors in NOD/SCID mice show that transplanted A549-Snail cells are capable of tumorigenesis. Data are shown as mean ± standard deviation (N = 5).

The metastatic potentials of A549-Snail and A459-vector cells were evaluated *in vivo* as follows. Both A549-Snail and A549-vector cells were administered to 4–6-week-old BALB/c mice by lateral vein injection. After 40 days, the numbers of metastatic colonies on the lung surface were counted. Compared to mice injected with A549-vector cells, mice injected with A549-Snail cells exhibited a remarkable increase in the number of metastatic colonies on the lung surface (Figure 3A), indicating that aggressive metastatic capacity is associated with Snail-induced EMT in A549-Snail cells *in vivo*.

To evaluate tumorigenicity *in vivo*, 1 × 10^4^ and 5 × 10^5^ A549-Snail cells were subcutaneously injected into 5 and 3 NOD/SCID mice, respectively. Ninety days later, the incidence of solid tumor formation was 100%. However, in the A549-vector–treated group, only 1 mouse developed a solid tumor after treatment with 5 × 10^5^ cells (Table 2). Furthermore, in the A549-Snail–treated group, the average tumor size was bigger than that in the A549-vector–treated group (Figure 3B). These data reveal that both EMT-mediated metastasis and CSC-mediated tumorigenicity are enhanced by Snail overexpression.

**Table 2:** The *in vivo* tumorigencity of A549-vector and A549-Snail

Cell type	Seeding density	Incidence of tested mice brought solid tumor
A549-vector	1 × 104	0/5
A549-Snail	1 × 104	5/5
A549-vector	1 × 105	1/3
A549-Snail	1 × 105	3/3

### Snail overexpression induces stem cell-like signatures during EMT

**Figure 4:**
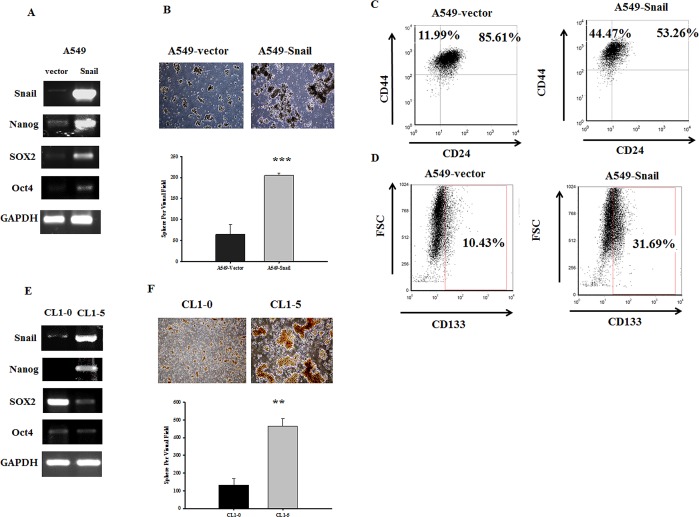
Snail overexpression induces stem cell-like signatures during the epithelial–mesenchymal transition (A/E) The mRNA expression of stemness genes (*Oct4*, *Sox2*, *Nanog*) was higher in A549 cells overexpressing Snail (A549-Snail cells) than in A549 cells expressing empty vector (A549-vector cells). However, only Nanog expression was higher in CL1-5 cells than in CL1-0 cells. (B/F) The number of spheroid-like bodies formed was significantly higher in Snail-expressing cells; ***p < 0.001 indicates statistical significance as compared to the control. (C/D/G/H) Cell-surface markers (CD24, CD44, and CD133) were analyzed by flow cytometry as described in the Methods section. Increases in the CD44^high^/CD24^low^ subpopulation (C) and the surface expression of CD133 (D) were found in A549-Snail cells as compared to the A549-vector cells.

Expression of stemness genes was also examined in A549-Snail cells. Stemness genes (e.g., *Sox2*, *Oct4*, and *Nanog*) were expressed at a low level or not expressed in A549-vector cells but were obviously induced in A549-Snail cells. *Nanog* was also expressed in CL-15 cells but not *Sox2* and *Oct4* (Figure 4A/4E). We also found that Snail expression is associated with an increase in the number of spheroid-like bodies formed (Figure 4B, F). In addition, using flow cytometry it was possible to examine cells for the presence of a stem cell-like population with a CD44^high^/CD24^low^ phenotype. The CD44^high^/CD24^low^ (CD44, 11.99% versus 44.47%; CD24, 85.61% versus 53.26%) phenotype occurred more frequently in A549-Snail cells than in A549-vector cells (Figure 3C). In addition, cell-surface expression of CD133 (a biomarker of CSCs) was increased threefold in A549-Snail cells (Figure 3D). These data demonstrate the crucial role of Snail in triggering stem cell-like phenotypes via Nanog expression.

### Snail and Nanog are highly expressed in NSCLC tissue biopsies

**Figure 5:**
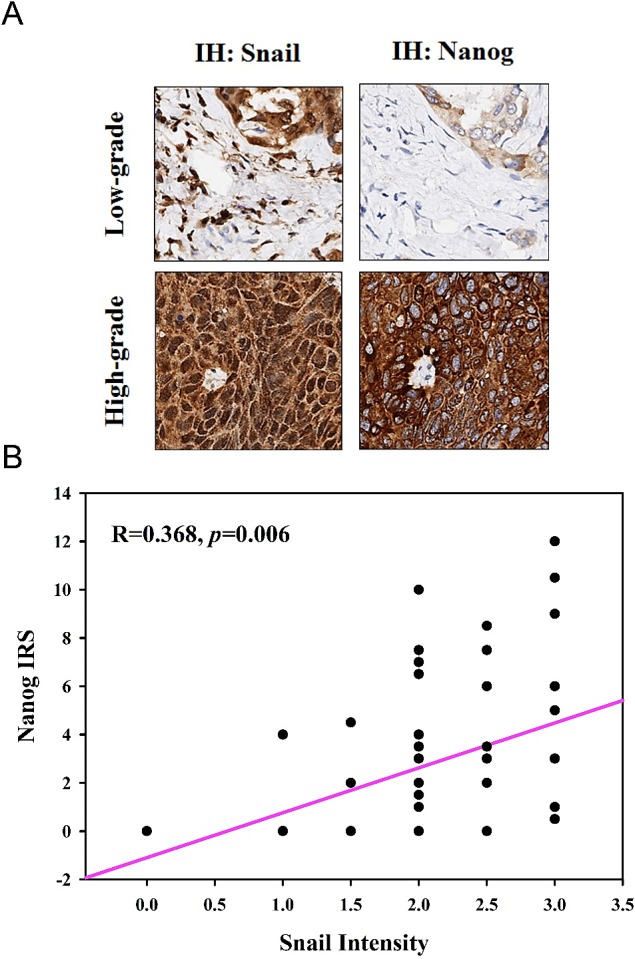
The expression of Snail and Nanog are highly correlated in lung cancer tissues (A) Representative specimens of low-grade and high-grade lung tumors immunostained using antibodies specific to Snail and Nanog. (B) Positive correlation between levels of Snail and Nanog in lung tumors, and the level of Nanog as a novel prognostic marker for lung cancer patients.

We then examined Nanog expression in 55 NSCLC tissue biopsies. Representative images show that Snail and Nanog were expressed at low levels in low-grade tumors. However, Snail and Nanog were highly expressed in high-grade tumors (Figure 5, Table 3).

**Table 3:** Correlation between Snail, Nanog expression and clinicopathological characteristics of lung cancer

	Snail Expression			
	n	Positive (%)	Negative (%)	P Value
Total	55	34 (62)	21(38)	
Nanog				
Positive	22	18	4	0.0126
Negative	33	16	17	

### PI-3 kinase/Akt activation and GSK3*β* inactivation are required for Snail-induced Nanog expression

**Figure 6:**
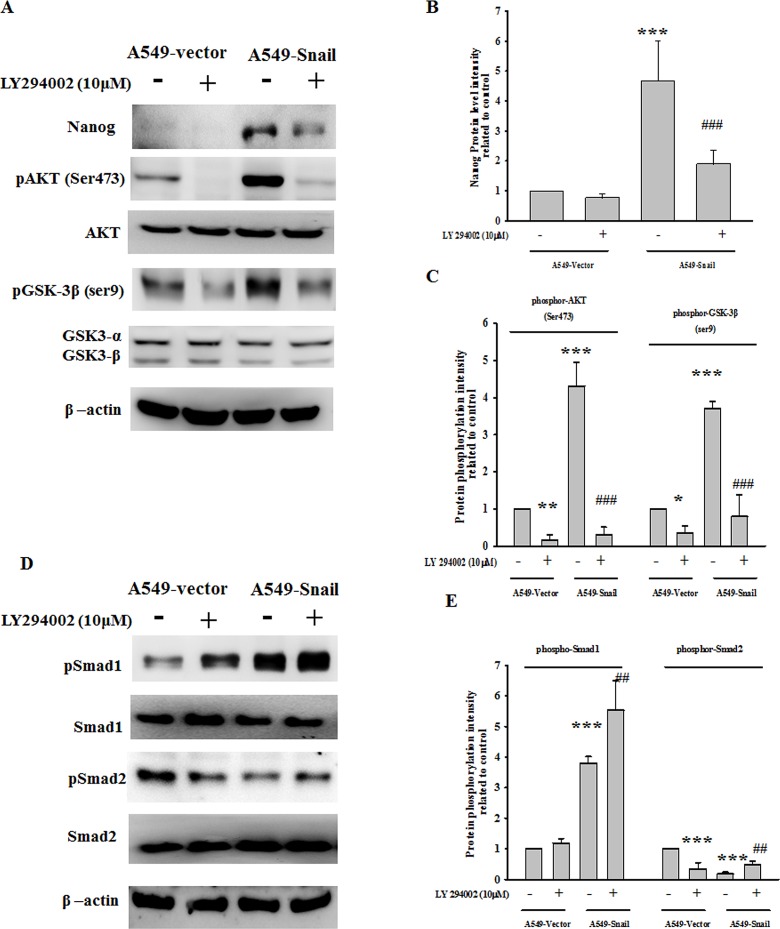
PI-3 kinase/Akt activation and GSK3β inactivation are required for Snail-induced Nanog expression (A) Remarkable activation of Akt (Ser-473 phosphorylation) and inactivation of GSK3β (Ser-9 phosphorylation) are found in A549 cells overexpressing Snail (A549-Snail cells) as compared to A549 cells expressing empty vector (A549-vector cells). Both Akt and GSK3β phosphorylation could be blocked by LY294002 pretreatment (10 μM; 1 h). In addition, Snail-induced Nanog expression was blocked by LY294002 pretreatment. (B/C) Data were quantified by densitometric analysis and expressed as the mean ± standard deviation from at least 3 independent experiments. (D) Remarkable activation of Smad1 and inactivation of Smad2 were found in the A549-Snail cells as compared to the A549-vector cells. Neither Smad1 phosphorylation nor Smad2 dephosphorylation was affected by LY294002 pretreatment. (E) Data were quantified by densitometric analysis and expressed as the mean ± standard deviation from at least 3 independent experiments; *p < 0.05, **p< 0.01, and ***p < 0.001 indicate statistical significance as compared to the A549-vector cells; ##p < 0.01 and ###p < 0.001 indicate statistical significance as compared to the A549-Snail cells.

As well as the mechanisms underlying Snail-induced EMT, the signals required for Snail-mediated Nanog expression remain unclear. In this study, activation of Akt (Ser-473 phosphorylation) and inactivation of GSK3β (Ser-9 phosphorylation) were found in A549-Snail cells but not in A549-vector cells (Figure 6A/6B), whereas the phosphorylation of ERK and STAT3 remained unaffected (Supplementary Figure 2A). Neither Akt nor GSK3β was phosphorylated when A549-Snail cells were coincubated with LY294002 (a PI-3 kinase inhibitor; 10 μM). These data indicate that Snail can activate PI-3 kinase/Akt, resulting in GSK3β inactivation and degradation. In addition, Nanog was not expressed in A549-Snail cells treated with LY294002 (Figure 6A/6C), whereas the STAT3 inhibitor WP-1066 (10 μM) had no effect on Nanog expression (data not shown).

### Smad1 is an upstream factor regulating Snail-induced PI-3 kinase/Akt and Nanog expression

**Figure 7:**
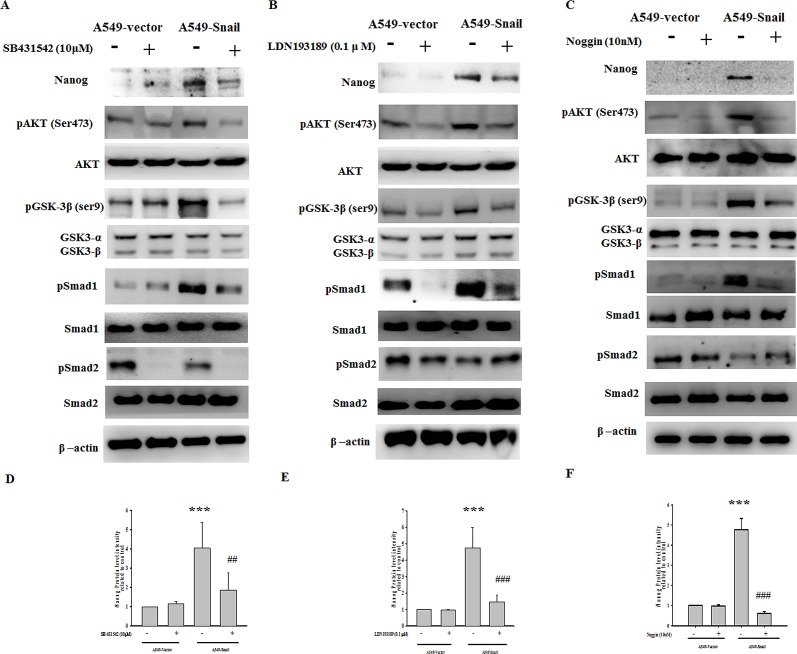
Snail-induced Nanog expression is regulated by Smad1/Akt/GSK3β pathway activation Snail-induced Akt and Smad1 activation and GSK3β inactivation was inhibited by SB431542 (A), LDN193189 (B), and Noggin (C) pretreatment. In addition, Snail-induced Nanog expression was prevented by SB431542, LDN193189, and Noggin pretreatment. (D/E/F) Data were quantified by densitometric analysis and expressed as the mean ± standard deviation from at least 3 independent experiments; ***p < 0.001 indicates statistical significance as compared to the A549-vector cells; ##p < 0.01 and ###p < 0.001 indicate statistical significance as compared to the A549-Snail cells.

Both TGF-β (which activates type I receptors or activin-receptor-like kinases [ALKs]) and BMPs (which activate type II receptors such as ActR-IIA, ActR-IIB, BMPR-II, AMHR-II, and TGFβR-II) play crucial roles in orchestrating EMT in various epithelial tissues. TGF-β can activate ALK4/5/7, causing Smad2/3 phosphorylation, whereas BMPs phosphorylate ALK1/2/3/6 and Smad1/5/8 [13-15]. In this study, activation of Smad1 and inactivation of Smad2 were found in A549-Snail cells but not in A549-vector cells. (Supplementary Figure 2B) Neither Smad1 phosphorylation nor Smad2 dephosphorylation was affected when A549-Snail cells were coincubated with LY294002 (Figure 6D/6E). In contrast, coincubation with SB431542 (a TGF-β receptor inhibitor; 10 μM), LDN193189 (a BMP receptor inhibitor; 0.1 μM) or Noggin (a BMP inhibitor; 10nM) could suppress Snail-induced Akt and Smad1 activation and GSK-3β inactivation (Figure 7 and Supplementary Figure 3).

In addition, inactivation of TGF-β receptor and BMP receptor signals decreased the level of Snail-induced Nanog expression (Figure 7 and Supplementary Figure 3). SB431542, LDN193189, or Noggin did not significantly affect Snail-induced Smad2 dephosphorylation (Figure 7 and Supplementary Figure 3), suggesting that the Snail/Smad2 signal is not involved in EMT or the regulation of Nanog expression. To confirm the involvement of Smad1/Akt/GSK3β signaling on Snail-induced EMT-associated anticancer drug resistance, cells were cotreated with cisplatin and LY294002, SB431542, or LDN193189. Cotreatment with LY294002, SB431542, or LDN193189 significantly reduced Snail-induced EMT-associated anticancer drug resistance (Supplementary Figure 4).

### Reduction in Smad1-Akt-GSK3β signaling and EMT phenotypic expression in Snail-silenced CL1-5 cells

**Figure 8:**
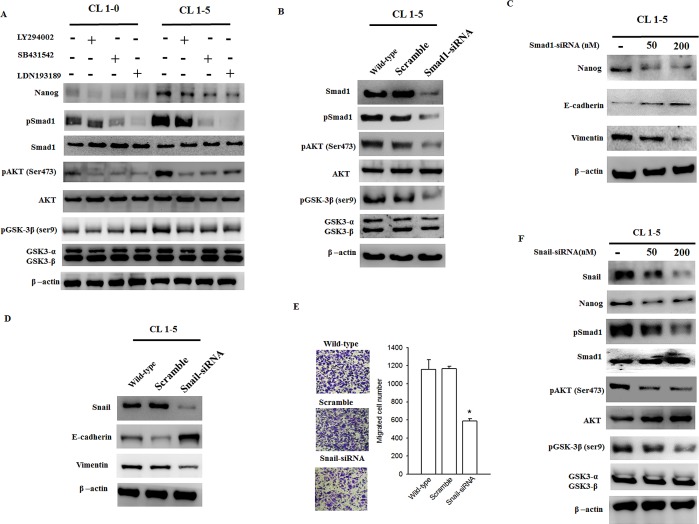
The Smad1/Akt/GSK3β pathway is consistently activated in CL1-5 cells but is downregulated in Snail-silenced CL1-5 cells (A) CL1-5 cells, which endogenously express Snail, exhibited greater activation of the Smad1/Akt/GSK3β pathway and upregulated Nanog expression in comparison to CL1-0 cells. Both SB431542 and LDN193189 decreased activation of the Smad1/Akt/GSK3β pathway and downregulated Nanog expression in CL1-5 cells. Unlike Akt and GSK3β phosphorylation, which was completely inhibited by LY294002, Smad1 phosphorylation did not respond to LY294002. (B/C) Scramble and Smad1 siRNA were expressed in CL1-5 cells for 40 h. The Smad1 siRNA fully suppressed the Smad1/Akt/GSK3β pathway as well as Nanog expression. In addition, the expression of mesenchymal markers decreased, but the expression of epithelial markers increased in Smad-silenced CL1-5 cells as compared to CL1-0 cells. (D) The endogenously expressed Snail was silenced in CL1-5 cells. An increase in E-cadherin and a decrease in vimentin expression were found in Snail-silenced CL1-5 cells. (E) In addition, a reduction in the number of migrating cells was observed in Snail-silenced CL1-5 cells as compared to the control cells (transfected with Scramble siRNA); ***p < 0.001 indicates statistical significance as compared to the control. (F) Either a decrease in the level of activation of the Smad1/Akt/GSK3β pathway or a reduction in Nanog expression was found in Snail-silenced CL1-5 cells.

Nanog expression and phosphorylation of Smad1, Akt (Ser-473), and GSK3β (Ser-9) were enhanced in CL1-5 cells as compared to CL1-0 cells (Figure 8A), which may explain the potent metastatic profile of CL1-5 cells. Moreover, SB431542 or LDN193189 fully suppressed Smad1, Akt, and GSK3β phosphorylation in CL1-5 cells. Unlike Akt and GSK3β phosphorylation, which were completely inhibited by LY294002, Smad1 phosphorylation did not respond to LY294002, suggesting that Smad1 is an upstream factor for Snail-induced Akt activation (or GSK3β inactivation) (Figure 8A). Inhibition of Smad1 by siRNA markedly decreased the expression of Akt and GSK3β phosphorylation and repression of Nanog expression in CL1-5 cells (Figure 8B, C). Endogenous Snail expression was silenced by Snail siRNA transfection in CL1-5 cells but not in sham-treated cells. Snail-silenced CL1-5 cells showed increased E-cadherin expression and decreased vimentin expression (Figure 8D), as well as a reduction in the number of migrating cells (Figure 8E) in comparison to the control group. In addition, Snail-silenced CL1-5 cells showed significantly increased sensitivity to drugs such as cisplatin or LBH589 in comparison to the control group (Supplementary Figure 5). Moreover, a decrease in Smad1/Akt/GSK3β pathway signaling and Nanog induction were found in Snail-silenced CL1-5 cells (Figure 8F). These data strongly indicate that Snail induces EMT via Nanog expression and the Smad1/Akt/GSK3β signaling pathway.

## DISCUSSION

In vertebrates, EMT is an essential process for embryonic development. Mesenchymal traits (fibroblast-like morphology, intercellular junction disruption, reduction of apicobasal polarity, cytoskeleton reorganization, etc.) enable cells to migrate to specific targets in the embryo, where they undergo differentiation. In cancer biology, the initiation of metastasis is phenotypically similar to EMT. It has been reported that hypoxia [16], 2,3,7,8-tetrachlorodibenzo-p-dioxin [17], nicotine [18], osteopontin [19], and TGF-β [9] induce EMT by increasing the expression of mesenchymal genes (such as vimentin, fibronectin, and integrins). Upregulation of Snail is highly correlated with these inducers, and in cancerous cells, exposure to dihydrotestosterone and TGF-β has been shown to induce EMT via Snail activation [20]. In addition, the involvement of other developmental factors (such as Slug, ZEB, FOXC, and Twist) has also been reported [21-23]. Here, we found that events related to epithelial property loss occurred in parallel to the gain of mesenchymal markers (notably, an increase in vimentin and N-cadherin expression) in Snail-expressing cells (Figure 2). The expression of these markers decreased when Snail expression was silenced by RNA interference (RNAi) (Figure 8). Other studies have also shown that RNAi inhibition of Snail decreased metastasis in subcutaneously injected models [24]. These data support the crucial role of the Snail protein in triggering EMT.

Snail protein is absent from normal immortalized prostate epithelial cells but is expressed in the malignant LNCaP and DU145 cell lines, which are characterized by high migration. Moreover, the migratory potential of these cells was decreased by Snail siRNA treatment [25]. Clinically, Snail overexpression correlates significantly with lymph node [26] metastasis, histologic grade, and TNM stage [27]; however, factors resulting in the development of CSC-like properties have not been fully identified. A recent study showed that suppression of miR-34 caused EMT and promoted stemness via the induction of Snail [28]. miR-34 has been shown to be highly induced by the tumor suppressor p53 and to directly inhibit the expression of CD44 [29, 30]. Snail has also been reported to repress the expression of p53 and protect cancer cells from cell death [31, 32]. Since CD44 positivity is associated with chemoresistance, allowing CD44-positive cells to escape and regrow can cause relapse and metastasis [33]. In this study, we have shown that ectopic expression of Snail in A549 cells increases the CD44^high^/CD24^low^ subpopulation and enhances surface expression of CD133 (Figure 4), which has been recognized as a CSC marker [34]. Furthermore, these Snail-overexpressing cells exhibited strong chemoresistance (Figure 2), which is a major clinical property defining CSCs [35]. Taken together, these results indicate a relationship between Snail, EMT, and cancer stemness.

Previous studies have defined a core transcriptional program for “stemness” through increased expression of stem cell-related stemness genes, indicating the remarkable plasticity of stem-cell fates. Further evidence suggests that the Oct4–Sox2 complex, at least in part, is involved in the regulation of Nanog expression [36]. In this study, Snail induced the transactivation of Nanog, Sox2, and Oct4 in A549-Snail cells but not in CL1-5 cells (Figure 4). Nanog is a transcription factor that is specifically active in embryonic stem cells. Recent studies have shown the pivotal role of Nanog in tumorigenesis of various tissues [37-39] and that Snail-positive tumors are correlated with tumor malignancy and recurrence [40-42]. In this study, we confirmed this correlation in 55 lung cancer tissue specimens and showed that Nanog expression is correlated with Snail expression, as well as patient age and tumor type, staging and malignancy (Tables 2 and 3).

Although it is clear that Snail induces Nanog expression via EMT to generate cancer stem cell-like characteristics, the signals downstream of Snail overexpression have not yet been elucidated. Oncogenic pathways such as those mediated by TGF-β, Src, Ras, PI-3 kinase/Akt, Wnt/β-catenin, Notch, nuclear factor-kappa B, activating transcription factor 2, and Hedgehog may be associated with EMT [43]. Of these, TGF-β signaling is a major pathways involved in EMT. It may directly or indirectly promote EMT via Wnt/β-catenin signaling or protein kinase A activation. TGF-β has been shown to affect EMT through the degradation of PIAS1 in epithelial cells. It has been suggested that PIAS1 may be a key regulator of TGF-β induced EMT [44] and metastasis [45]. Ras-MAPKs have been shown to elevate endogenous Snail, Slug, and Twist expression [46], and Snail is known to repress the expression of maspin or E-cadherin [47]. Importantly, Snail expression can promote a motile phenotype and enhance invasion through the basal membrane. The** phenotypic changes associated with Snail expression include both increased cell motility and the production of extracellular matrix-degrading enzymes such as matrix metalloproteinases (MMPs) and are always accompanied by the disruption of E-cadherin-mediated cell–cell adhesion [48]. As such, a functional link between E-cadherin and MMPs has been established. In addition, a recent study has shown a positive correlation between loss of E-cadherin and ERK activation, which promotes invasion via ZEB1/MMP2 axis [49]. However, in our study, MAPK phosphorylation did not change in Snail-overexpressing cells (Supplementary Figure 2A), and neither E-cadherin expression nor migratory potential was changed in Snail-overexpressing cells pretreated with MAPK inhibitors, as compared to controls (data not shown).

In this study, we found that Snail-induced expression of the “stemness” gene Nanog could be inhibited by LY294002, SB431542, LDN193189, and Noggin, suggesting the involvement of the Smad1/Akt/GSK3β pathway in stem cell-like transformation. In this study, we also observed hyperactivation of Akt and Smad1 and inactivation of GSK3β (phosphorylated at Ser-9) and Smad2 in Snail-overexpressing EMT cells. Snail-induced Akt hyperactivation and GSK3β inactivation could be inhibited by LY294002, SB431542 (a TGF-β receptor inhibitor), LDN193189 (a BMP receptor inhibitor) or Noggin (a BMP inhibitor) pretreatment, whereas Smad1 phosphorylation was not affected by LY294002 (Figures 4 and 5). Intense Smad1 phosphorylation has been observed not only in the Snail-overexpressing cells in our study but also in metastatic tumor tissues derived from breast cancer [50]. These data provide evidence for the functional role of Smad1 in EMT stem cell-like transformation. Snail-mediated upregulation of Nanog expression via the activation of the Smad1/Akt/GSK3β pathway has also been identified in another potently metastatic NSCLC cell line, CL1-5, which expresses Snail endogenously (Figure 8).

**Figure 9:**
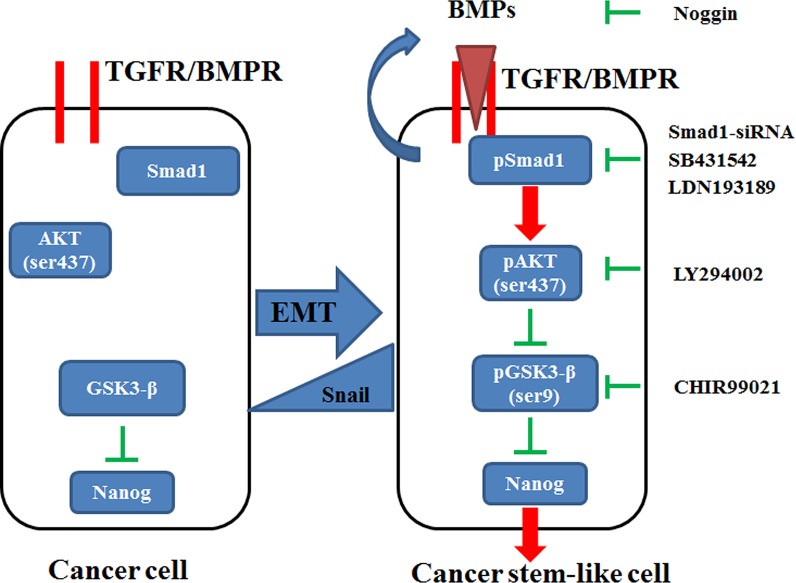
Diagram showing the induction of stem cell-like properties by Snail via the activation of the Smad1/Akt/GSK3β pathway and subsequent upregulation of Nanog expression

In conclusion, this study shows that either endogenous or ectopic Snail protein increases Nanog expression and activates the Smad1/Akt/GSK3β pathway. Pretreatment of the cells with LY294002, SB431542, LDN193189, or Noggin inhibits Snail-mediated Nanog induction, resulting in the retrieval of EMT and stem cell-like progression. To the best of our knowledge, this is the first study to illustrate the effects of Snail protein on Nanog expression and CSC-like transformation. In addition, this study is the first to provide evidence that the Smad1/Akt/GSK3β pathway is linked to Snail-induced Nanog expression and CSC-like transformation in NSCLC cells (Figure 9). These findings will be helpful to explain how cancer cells maintain their stemness and aggressiveness. Better understanding of these signals will aid in the development of new chemotherapeutic targets and drug discovery.

## METHODS

### Cell culture and chemicals

The NSCLC cell line A549 was purchased from the American Type Culture Collection (Manassas, VA, USA). The CL1-0 and CL1-5 cell lines were kindly provided by Dr. Chen Huei-Wen (Institute of Toxicology, National Taiwan University, Taipei, Taiwan). A549 and CL1-0/CL1-5 cells were routinely maintained in Dulbecco's Modified Eagle's Medium (DMEM) and Roswell Park Memorial Institute (RPMI) 1640 medium, respectively, with 10% heat-inactivated fetal bovine serum.

The chemical inhibitors (LY294002, PD98059, WP1066, and SB431542) were obtained from Calbiochem (San Diego, CA, USA). LDN193189 was obtained from Axon Medchem (Postbus, Groningen, The Netherlands). The maximal final concentration of dimethyl sulfoxide in the medium was 0.1%, which did not exhibit toxicity.

### Human Snail gene overexpression and silencing

Overexpression of the human Snail protein was achieved by transfection with pcDNA3.1-h*Snail*, which contained the full-length *Snail* gene (Gene ID 6615) and was constructed using the pcDNA™3.1/V5-His TOPO® TA expression kit (Invitrogen). Transfection was performed using Turbofect™ (Fermentas). Successfully transfected cells were selected via a 24-h incubation in culture medium supplemented with G418 (350 μg/mL). For CL1-5 cells, transfection was performed with 50 nM control (Scramble) siRNA (sc-37007; Santa Cruz), 50 nM Snail-specific siRNA (sc-38398; Santa Cruz) or 50 nM Smad1-specific siRNA (sc-29483; Santa Cruz) using Turbofect™.

### Messenger RNA expression analysis using reverse transcription-polymerase chain reaction

Total RNA was extracted using TriPure reagent (Roche, Rotkreuz, Switzerland) according to the manufacturer's protocol. First-strand complementary cDNA was generated by reverse transcription using MMLV high-performance reverse transcriptase (Epicentre, Madison, WI, USA), and oligo (dT16–18) primers in a 20 μL reaction containing 3–5 μg of total RNA. The primers used in this study are summarized in Supplementary Table 1.

### Immunoblot analysis

A standard protocol was used for immunoblot analysis [51]. The commercial antibodies used in this study are listed in Supplementary Table 2. For chemiluminescent detection, blots were incubated with horseradish peroxidase-conjugated secondary antibodies (1:5000; Cayman, Ann Arbor, MI, USA) at room temperature, followed by enhanced chemiluminescence detection according to the manufacturer's protocol (Millipore, Billerica, MA, USA). All blots were also immunoblotted for β-actin (Sigma, St. Louis, MO, USA) to demonstrate equal loading of protein samples. All immunoblotting experiments were repeated at least 3 times.

### Lung colonization assay and xenograft tumor formation assay

The *in vivo* metastatic potential and tumorigenic abilities of A549 cells (wild-type or Snail-overexpressing) were measured using the lung colonization assay and xenograft tumor formation assay [51]. BALB/c mice and NOD/SCID mice were obtained from the National Taiwan University Animal Center and housed aseptically in its animal facilities.

For the lung colonization assay, a single-cell suspension (1 × 10^6^ cells) of A549 cells (wild-type or Snail-overexpressing) was prepared in 0.1 mL serum-free DMEM and then injected into the tail vein of 4–6-week-old BALB/c mice. Forty days later, the mice were anesthetized with isoflurane and sacrificed. The lungs were fixed with Bouin's solution, and metastatic colonies on the lung surface were counted macroscopically.

For the xenograft assay, cell suspensions (5 × 10^5^ and 1 × 10^4^ cells/0.1 mL DMEM) were injected subcutaneously into the left and right sides, respectively, of 4– 6-week-old NOD/SCID mice. Resulting tumors were measured by using calipers to determine the 2 orthogonal external diameters. All mice were anesthetized and sacrificed on day 90 after injection.

### Immunohistochemical analysis

The human lung cancer (LUC1501) tissue microarray used in this study was purchased from US Biomax Inc. (Rockville, MD, USA). The slides were stained for Snail (Abcam; 1:250 dilution) and Nanog (Epitomics; 1:200 dilution) at the Department of Pathology, National Taiwan University Hospital. The tissue specimen grades were confirmed by a pathologist and included 3 normal lung tissues, 14 adenocarcinomas, 31 squamous cell carcinomas, 7 adenosquamous carcinomas, and 3 metastatic adenocarcinomas. The immunoreactive score was calculated by multiplying the staining intensity score by the percent positivity [52], which were determined by an experienced pathologist (Dr. Yi-Jen Peng, Department of Pathology, Tri-Service General Hospital, National Defense Medical Center, Taipei, Taiwan).

### Statistical analysis

All data are expressed as the mean ± standard deviation from at least 3 independent experiments (n > 3). Statistically significant differences among groups were determined using 1-way analysis of variance (ANOVA), and *p* values** <0.05 were considered to indicate statistical significance. Statistical correlations between the results of immunohistochemical analyses were calculated using the χ^2^ test. SigmaPlot software (Systat Software, Inc., CA, USA) was used to assess linear regression in analyzing the relationship between Snail and Nanog immunostaining scores.

## SUPPLEMENTARY TABLES AND FIGURES



## Abbreviations

non-small cell lung cancer (NSCLC)
epithelial–mesenchymal transition (EMT)
cancer stem cell (CSC)
phosphatidylinositol-3 kinase (PI-3 kinase)
glycogen synthase kinase-3 beta (GSK3β)
transforming growth factor beta (TGF-β)
bone morphogenetic protein (BMP)
