# Supplementary material for: Snail regulates Nanog status during the epithelial–mesenchymal transition via the Smad1/Akt/GSK3β signaling pathway in non-small-cell lung cancer
Source: Oncotarget. 2014 May 26;5(11):3880–94. doi: 10.18632/oncotarget.2006 (PMC4116528; doi:10.18632/oncotarget.2006)
Supplement: Supplementary file 1 [file oncotarget-05-3880-s001.pdf]

Snail regulates Nanog status during the epithelial–mesenchymal transition via the Smad1/Akt/GSK3β signaling pathway in non-small-cell lung cancer

Supplementary Table 1. The primer sets used in this study.

Gene	Primer sense	Primer antisense
**Snail**	ACTATGCCGCGCTCTTTCCT	AGTCCTGTGGGGCTGATGTG
**Oct4**	GTCCGAGTGTGGTTCTGTA	CTCAGTTTGAATGCATGGGA
**Nanog**	CAAAGGCAAACAACCCACTT	TCTGCTGGAGGCTGAGGTAT
**Sox2**	ATGGGTTCGGTGGTCAAGT	GCTCTGGTAGTGCTGGGACA
**MMP-2**	GGCCCTGTCACTCCTGAGAT	GGCATCCAGGTTATCGGGGA
**MMP-14**	GATGTTTGTCTTCAAGGAGCG	CCTTCAGCTTCTGGTTGTTGA
**GAPDH**	ACCCAGAAGACTGTGGATGG	CAGTGAGCTTCCCGTTCAG

Supplementary table 2: Antibodies used in this study

Name	Source	Manufacture
Snail	Rabbit	Cell signaling
Snail (IHC)	Rabbit	Abcam
E-cadherin	Rabbit	Cell signaling
Occludin	Rabbit	Genetex
N-cadherin	Rabbit	Epitomics
Vimentin	Rabbit	GenScript
Fibronectin	Rabbit	Cell signaling
pAkt (Ser473)	Rabbit	Epitomics
Akt	Rabbit	Santa Cruz
pGSK-3β (Ser9)	Rabbit	Epitomics
GSK-3β	Rabbit	Epitomics
Nanog	Rabbit	Cell signaling
Nanog (IHC)	Rabbit	Epitomics
pSmad1/5	Rabbit	Cell signaling
Smad1	Rabbit	Cell signaling
pSmad2	Rabbit	Cell signaling
Smad2	Rabbit	Cell signaling
β–actin	Mouse	Sigma

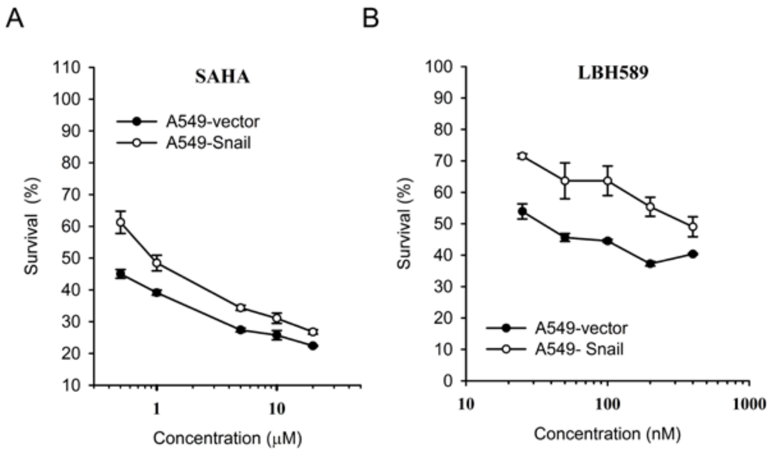

Supplemental Figure 1: A549-Snail are resistant to chemotherapy. A549-vector and A549-Snail cell were treated with HDAC inhibitor, SAHA or LBH589, then cell viability was assayed by MTT. Data shown are the mean ± S.D. of 3 independent experiments. The data proved A549-Snail was significantly resistant to SAHA and LBH589, in compared with A549-vector.

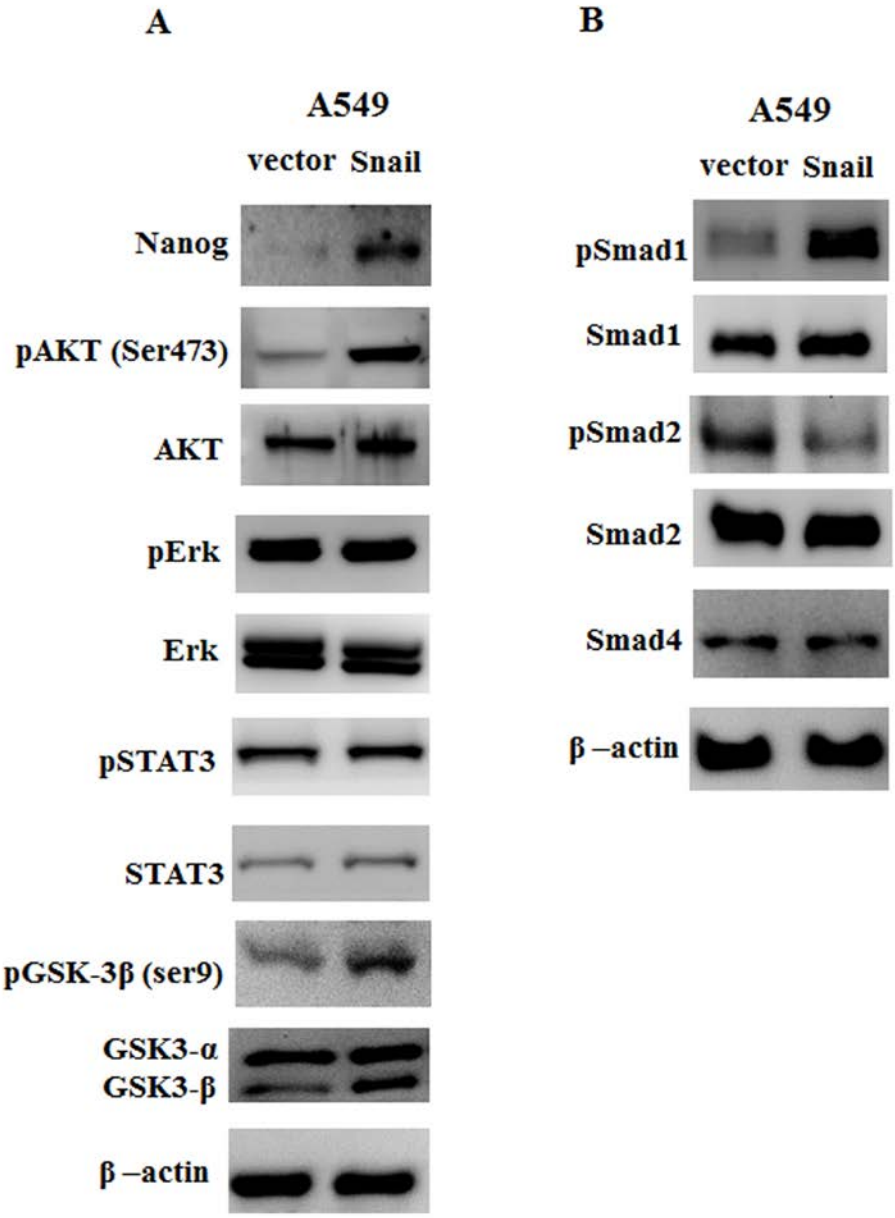

Supplemental Figure 2: Snail enhances phosphorylation of pAKT, pGSK-3β and Smad1, dephosphorylation of pSmad2 and the phosphorylation of pERK and pSTAT3 remained unaffected in A549-vector and A549-Snail cells. These data were analyzed by immune-blot as described in the ''Materials and Methods'' section.

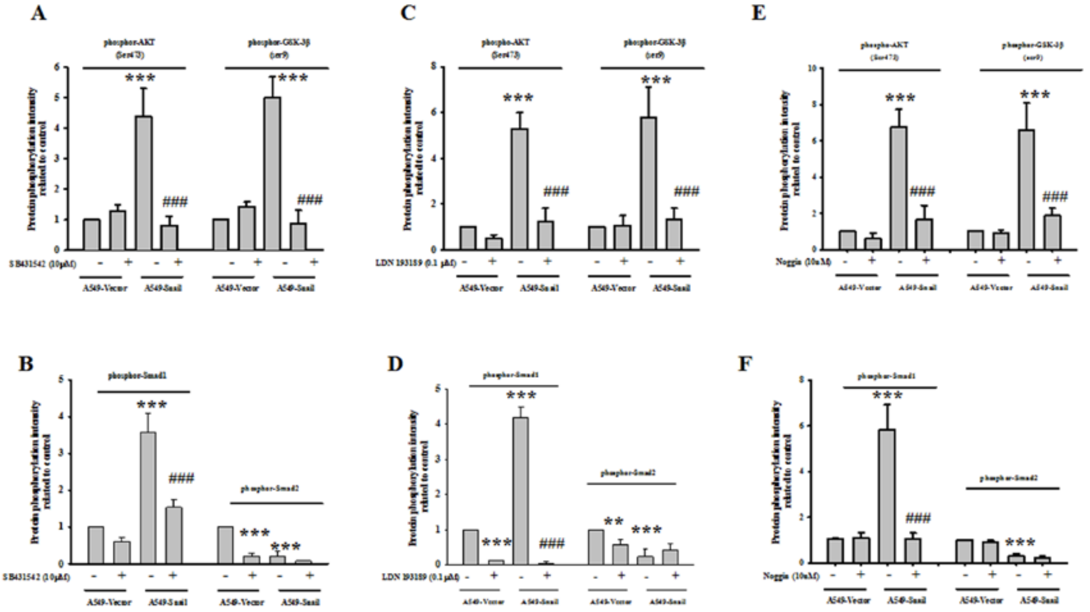
A
C
E
B
D
F
Protein phosphorylation intensity related to control
SB431542 (10μM)
LDN 193189 (0.1 μM)
Noggin (10nM)
A549-Vector
A549-Snail
***
###

Supplementary Figure 3: The quantified analysis of Snail-mediated Smad1/5-Akt-GSK3β axis activation (presented in Figure 5). TGF-β receptor inhibitor SB431542 (10 μM) reversed Snail-mediated Akt activation, GSK-3β inactivation (A), and Smad1 activation, whereas the Smad2 inactivation remained unaffected (B). (C/D/E/F) ALK inhibitor LDN193189 (0.1 μM) and BMP antagonist, Noggin, showed the similar effects. All data were quantified by densitometric analysis and expressed as mean ± S.D. from at least 3 independent experiments (**$p < 0.01$, and ***$p < 0.001$ indicate a statistically significant difference from the A549-vector control group; ###$p < 0.001$ indicate a statistically significant difference from the A549-Snail group). These data proved the activation of Smad1-Akt-GSK3β axis in Snail overexpressing cells.

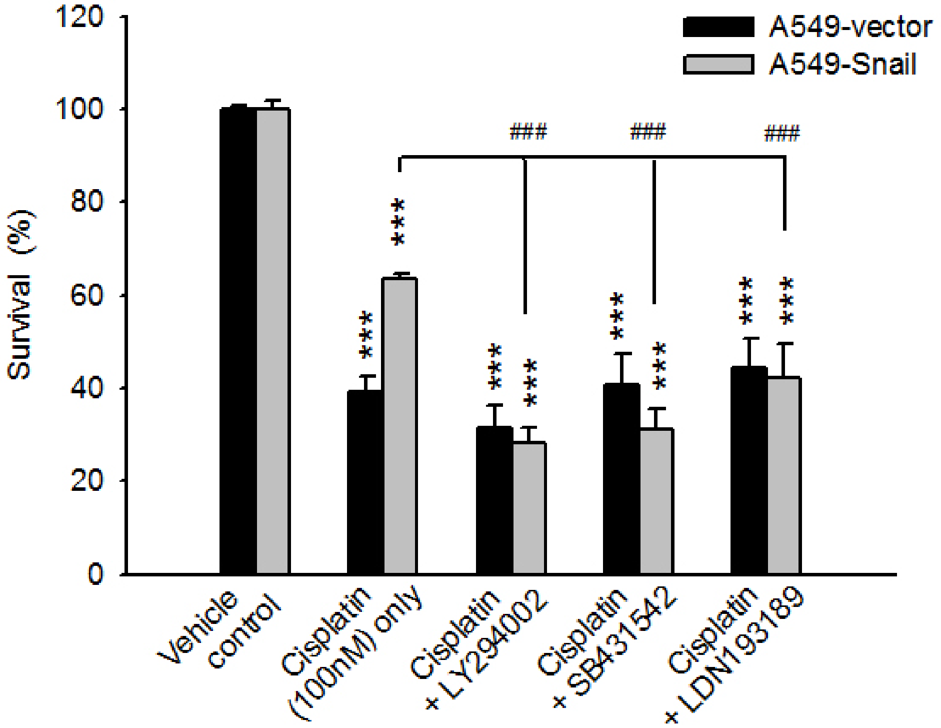

Supplementary Figure 4: The chemoresistance of A549-Snail was rescued by signaling inhibitors specific to Smad1-Akt-GSK3β axis. A549-vector and A549-Snail cell were pre-treated with LY294002, SB431542 or LDN193189, followed by a co-treatment with cisplatin. Then, cell viability was assayed by MTT. Data shown are the mean ± S.D. of 3 independent experiments (***p < 0.001 indicate a statistically significant difference from the vehicle control group; ###p < 0.001 indicate a statistically significant difference from the cisplatin treated group). The data proved the chemoresistance of A549-Snail was resulted from Snail-mediated Smad1/5-Akt-GSK3β axis activation.

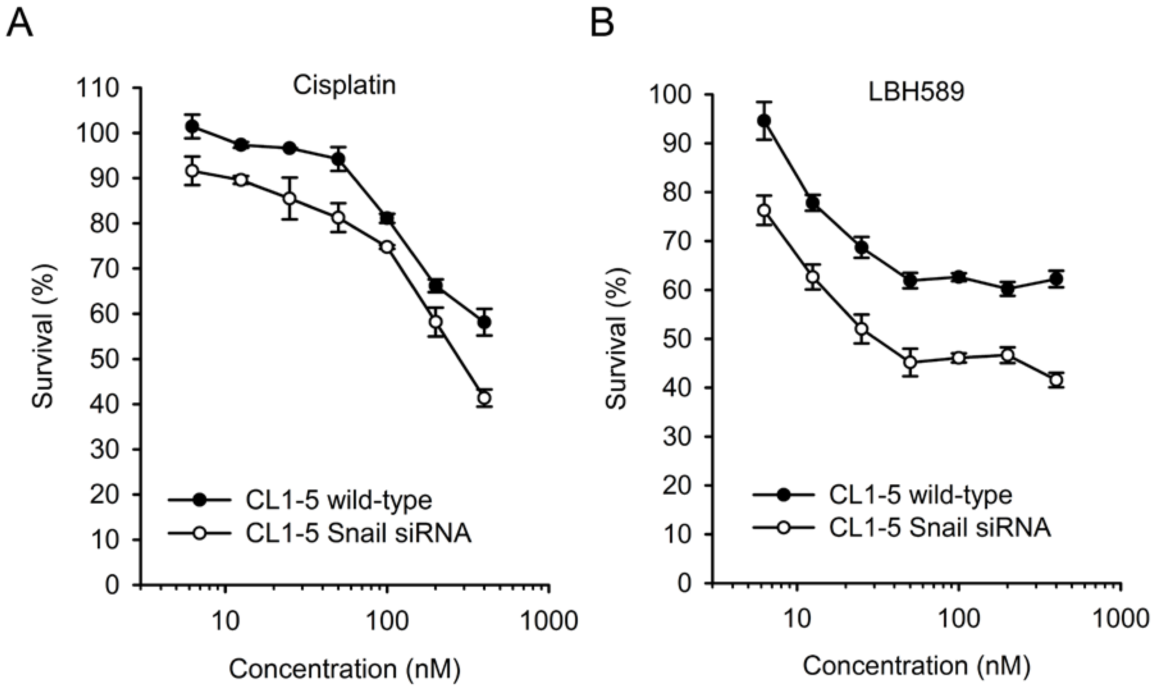

Supplementary Figure 5: The chemoresistance of CL1-5 (with endogenous Snail) was rescued by Snail down-regulation. CL1-5 and Snail-silenced CL1-5 were treated with cisplatin or LBH589, and then cell viability was assayed by MTT. Data shown are the mean ± S.D. of 3 independent experiments. The data showed the silence of endogenous Snail in CL1-5 cell sensitized CL1-5 to chemotherapy (cisplatin and LBH589).
